# The Role of Gene Alterations in the Pathogenesis of Polycystic Ovary Syndrome

**DOI:** 10.3390/jcm14103347

**Published:** 2025-05-12

**Authors:** Enrico Carmina

**Affiliations:** Endocrinology Unit, School of Medicine, University of Palermo, 90139 Palermo, Italy; enricocarmina28@gmail.com

**Keywords:** polycystic ovary syndrome (PCOS), gene candidate studies, GWAS, epigenetic factors, environmental factors, obesity, *DENND1A* gene, AMH

## Abstract

Family studies have shown that polycystic ovary syndrome (PCOS) has probable genetic transmission because of a high incidence of relatives who present clinical or biochemical characters of the syndrome. However, initial candidate gene studies were unsuccessful. Genome wide association studies (GWASs) have shown that at least 29 gene alterations are common in PCOS, but it has been calculated that the altered genes found by GWASs may represent only 10% of affected patients. Rare altered uncoding genes may explain the syndrome in an additional group of patients. In many other patients, the altered genes found by GWASs may represent a risk condition for the development of the syndrome, and new candidate gene studies have shown that some gene alterations that mainly concern androgen production may be common in PCOS. Finally, in most patients, epigenetic and environmental factors may be necessary to transform a risk condition into this common and important syndrome.

## 1. Family Studies and Candidate Gene Evaluation

Polycystic ovary syndrome (PCOS) is a common disorder in ovarian function that is characterized by a variable combination of hyperandrogenism, anovulation, and multi-follicular ovaries (with at least two factors needed for diagnosis) [1,2,3]. It is a very heterogeneous condition, and for many years, the etiopathogenesis of the syndrome has remained unclear.

Several years ago, studies on the families of individuals with PCOS suggested that this syndrome had a probable genetic transmission because of a high incidence of relatives presenting with clinical or biochemical characteristics of PCOS [4,5,6]. In fact, it was reported that 35% of mothers and 40% of sisters of 195 patients with PCOS were affected by PCOS themselves [4]. Similar data were reported in a study that found that 46% of sisters of women with PCOS were hyperandrogenic [5]. Finally, a large study based on the Netherlands twin registry reported that the syndrome is present in 70% of 1332 monozygotic twins but also in 38% of 1873 dizygotic or non-twin sister pairs [6]. This study confirmed that gene alterations are present in many patients with the syndrome but also suggested that the genetic component of PCOS is probably determined by a few genes.

However, studies on specific genes have generally been unsuccessful [7].

The candidate genes that have been studied include genes influencing different functions that are altered in the syndrome, in particular:

- Steroid metabolism;
- Insulin action;
- Gonadotropins;
- Obesity and fuel metabolism.

While cultures of theca cells from PCOS patients were found to produce exaggerated quantities of androstenedione [8], no specific genetic alterations to the enzymes involved in the steroidogenic process were demonstrated [7,9].

Several years ago, it was suggested that an abnormal serine phosphorylation of insulin receptor (maybe alteration of protein kinase C (PKC), a serine-threonine kinase) [10] is present in individuals with PCOS and causes the insulin resistance that is associated with the syndrome. However, more recent studies on the same group did not report this alteration [11], and genome-wide association studies did not find this genetic alteration in PCOS patients [12,13,14,15,16,17].

Similarly, in recent studies, other candidate gene alterations that have been posited as being important in the pathogenesis of PCOS were not confirmed. About 20 years ago, it was reported that PCOS patients presented an alteration in a gene on chromosome 19p13.2 (D19S884) that is part of the Fibrillin-3 gene (FBN3) [18]. However, this genetic alteration has not been confirmed in large studies like genome-wide association studies (GWASs) [12,13,14,15,16,17].

In conclusion, while the study of families of patients with PCOS has shown that most characteristics of the syndrome are present in relatives of the patients, suggesting that up to 70% of patients with PCOS may have the genetic transmission of the syndrome, for many years, the search for specific gene candidate alterations was unsuccessful, with the exception of some uncommon polymorphisms of the AntiMullerian hormone (AMH) gene.

## 2. Genome-Wide Association Studies

In 2011, a Chinese group published the first genome-wide association study (GWAS) in women with PCOS [12]. In this study, a completely different approach was used. The complete genome of women with PCOS was compared with the genome of the general population to try to identify differences in gene expression. The study included about 4000 women with PCOS, while the control group of women without PCOS was composed of more than 6000 individuals [12].

This initial study showed genetic differences in three chromosome areas: 2p16.3, 2p21, and 9q 33.3 [12]. The alterations in the 2p16.3 chromosome area concerned the luteinizing hormone/human choriogonadotropin (LH/hCG) receptor that is involved in ovulation and ovarian theca cell androgen production and transcription factor IIA (TFIIA) alpha and beta-like factors. No differences in follicle-stimulating hormone (FSH) molecules or receptors were found in this study. The alterations in the 2p21 chromosome area concerned the thyroid adenoma-associated (*THADA*) gene system, while the alterations in 9q33.3 were related to DENN (differentially expressed in normal and neoplastic cells) domain-containing protein 1A (*DENND1A)* (involved in endocytotic trafficking and clathrin-mediated endocytosis).

Further studies by the same group expanded their results, confirming the alterations in these three chromosome areas and suggesting the involvement of eight genetic loci [13].

Several other studies using the GWAS method followed, both in Europe and in the USA. These studies confirmed the findings of Chinese studies (alterations in the *DENND1A* and *THADA* single-nucleotide polymorphisms (SNPs)) but also identified some additional genetic alterations and, in particular, an alteration in the *FSHB* gene, which encodes the beta subunit of FSH [14,15,16].

More recently, a large study that included many research groups and performed a GWAS in 21,570 patients with PCOS has further enlarged the number of gene alterations that may be found in individuals with PCOS [17]. In total, 29 gene loci associated with PCOS have been identified through Chinese and European ancestry population studies [12,13,14,15,16,17].

The findings of these studies may be summarized according to the involved function: hyperandrogenism, chronic anovulation and polycystic ovaries, and hyperinsulinemia and insulin resistance.

### 2.1. Genetic Alterations That Are Linked to Hyperandrogenism

In individuals with PCOS, GWASs have shown that hyperandrogenism may depend on the following:

- The increased expression of the *DENND1A* gene, an important regulator of theca cells androgen biosynthesis [12,13,14,15,16,17];
- The increased expression of the *FSHB* gene, a gene that is involved in the production of the B chain of FSH [17];
- The increased expression of THADA [12,13,14,15,16,17];
- The increased expression of the LH receptor gene (LHCGR) in theca (and granulosa) cells [12,13,14,15,16,17].

The increased expression of the *DENND1A* gene seems particularly important in determining hyperandrogenism in PCOS [12,13,14,15,16,17,19,20,21,22]. It has been suggested that this genetic alteration increases the theca cell activity of Cytochrome P-450 17A1 (CYP17A1), thus increasing androgen production by the ovary [20,21,22]. It is probably a common genetic aspect of PCOS.

However, the increased expression of THADA and *FSHB*-altered genes may also play an important role in determining or increasing androgen excess in individuals with the syndrome [12,13,14,15,16,17].

Increased dehydroepiandrosterone sulfate (DHEAS) production (a characteristic that suggests increased adrenal androgen production) has been linked to the altered expression of the cytochrome P450 3 (CYP3) gene but only in one GWAS study [1].

### 2.2. Genetic Alterations That Are Linked to Chronic Anovulation and/or Polycystic Ovaries

Several genetic mechanisms may be operative and determine chronic anovulation and/or polycystic ovaries by different mechanisms:

(a) Influencing follicle recruitment and development

In PCOS patients, GWASs have shown several altered genes influencing recruitment and follicle development, but the most important seem to be the following:

- YAP1 (yes-associated-protein 1);
- ERBB4 (epidermal growth factor receptor 4);
- AMH.

The altered *YAP1* gene seems to be the most common mechanism, because this alteration was found in at least four GWASs, while the ERBB4 alteration was reported in at least three GWASs [12,13,14,15,16,17]. The altered *AMH* gene has been documented in only one GWAS [17].

(b) Increasing LH production and/or increasing LH/FSH ratio

An important role of altered gonadotrophin production with increased LH/FSH ratio in chronic anovulation in PCOS has been suggested by many studies. For many years, before the National Institutes of Health (NIH) and Rotterdam guidelines [1,23], an increased LH/FSH ratio was considered the main criterion for the diagnosis of PCOS. This diagnostic criterion was abandoned after the finding that many PCOS patients present with a normal LH and LH/FSH ratio [2,3].

GWASs have shown that altered genes that increase or alter FSH and LH production are common in individuals with PCOS. At least three altered genes regulating gonadotrophin secretion and action have been identified in PCOS:

- FSHB;
- LHR;
- FSHR.

The most common alteration concerns the *FSHB* gene, a gene that is involved in the production of the B chain of FSH. This alteration has been found in at least four GWASs [12,13,14,15]. The other alterations involve the receptor for LH (*LHCGR* gene) and the receptor for FSH (*FSHR* gene), which have been documented in at least two GWASs [12,13,14,15,16,17].

### 2.3. Genetic Factors That Are Linked to Hyperinsulinemia and Insulin Resistance

Hyperinsulinemia and insulin resistance are characteristics of PCOS that not only influence the metabolic characters of patients (and the increased diabetic risk) but, when severe, may determine anovulation [3]. In fact, it is well known that in obese PCOS patients, ovulatory cycles may be restarted via weight reduction [24,25,26,27].

Through GWASs, at least three altered genes regulating metabolism and insulin action have been identified in PCOS [12,13,14,15,16,17]:

- THADA;
- INSR (insulin receptor);
- HMGA2 (high-mobility group AT-hook 2).

Only *THADA* genes have been found to be altered in most GWASs, but in a large, recent GWAS, they were not associated with insulin or insulin resistance [17]. The other two genes have been found only in one GWAS, and therefore their role in the pathogenesis of insulin resistance in PCOS is unclear.

These data suggest that in most patients with PCOS, insulin resistance is not linked to specific or classic genetic alterations but may be the consequence of environmental factors acting on individuals with a genetic risk for the development of the syndrome. This hypothesis is supported by the finding that populations of PCOS patients living in different geographic areas present important differences in body weight and the severity of their insulin resistance [28,29,30].

In Table 1, the most common genetic alterations found by GWASs and their distributions in European or Chinese populations are reported [12,13,14,15,16,17].

## 3. Possible Interpretations of Genetic Alterations Found by GWASs

After the initial enthusiasm for the results of this new genetic methodology, it became clear that the results obtained via GWASs allowed for only a partial understanding of the mechanisms involved in the pathogenesis of PCOS.

In fact, it was calculated that the altered genes found by GWASs may represent only 10% of affected patients [31].

In addition, GWASs indicated same genetic loci in women with PCOS independently on their phenotypes [12,13,14,15,16,17]. Because the Rotterdam phenotypes of PCOS include a group of patients with very different clinical presentations (anovulatory and ovulatory women, lean and obese patients, patients with important alterations in metabolic patterns, and patients with no clinically relevant metabolic alterations, women with hyperandrogenism and women with normal androgen secretion) [3,32], it is probable that all these genetic alterations found in GWASs on large populations identify a small group of patients with the syndrome but mainly patients at risk for developing PCOS.

In conclusion, the pathogenetic mechanisms determining the clinical syndrome in the majority of patients and its heterogeneous presentation have not been fully clarified by GWASs.

## 4. New Candidate Gene Studies and the Role of Rare Genes in the Pathogenesis of PCOS

Recent studies have suggested that, similarly to the results reported for increased testosterone in the UK population [33], many uncommon different genetic alterations may be important in the pathogenesis of PCOS [31]. In fact, while GWASs are not able to uncover the rare gene alterations that may be involved in the pathogenesis of PCOS, gene alterations identified by GWASs have paved the way for new candidate gene studies.

Particularly important in the pathogenesis of the syndrome seem to be rare AMH and *DENDD1A* gene variants. In fact, at least thirty-seven rare variants of *AMH* genes have been found, and these variants may explain an additional 7–10% of PCOS patients [31,34,35,36]. These studies have also shown that altered AMH may affect the regulation of ovarian follicle development and increased AMH values during pregnancy may determine epigenetic consequences in the fetus, favoring the development of PCOS during the puberal age [34,35,36].

More important seem to be the variants of the *DENND1A* gene that have been found in up to 50% of families with PCOS [19,20,21,22,31,37], and these data confirm the central role of increased androgen production in PCOS.

Many other gene variants including genes that impair leptin signaling or the production of other adipokines or genes that may alter steroidogenesis have been reported [38], but in few samples, and their role remains unclear.

Interestingly, most of these rare genes are uncoding regulatory genes, that is, genes that regulate coding genes.

## 5. Integrated Models Including Genetic and Clinical Factors

Integrated models that include genetic and clinical factors and the use of machine learning methods may permit a better understanding of the pathogenesis of PCOS. Based on these models, it has been suggested that two main forms of PCOS exist: a metabolic subtype (characterized by a higher body mass index (BMI) and insulin levels) and a reproductive subtype (characterized by higher LH and sex hormone-binding globulin (SHBG) levels) [31]. Each PCOS subtype presents specific genetic associations.

The reproductive subtype is associated with a gene variant of type 1 AMH receptor (Bone morphogenetic protein receptor 1B (BMPR1B)) and an estrogen receptor coactivator (Positive regulatory domain 2 (PRDM2)). The *BMPR1B* gene regulates follicular development and androstenedione production.

The metabolic subtype may be linked to other genes [31].

An additional number of patients (the indeterminate subgroup) include patients that are not part of these two subgroups [31].

While the proposed names of the PCOS subtypes may not correspond to clinical patterns (all PCOS subtypes are reproductive because involve ovarian androgen production and follicle growth and regulation), this approach may represent an important step in our understanding of the pathogenesis of PCOS.

A recent study [39] has confirmed and expanded these results, indicating that the reproductive PCOS phenotype is associated with higher levels of AMH and a higher total follicular count while the metabolic subtype is associated with higher low-density lipoprotein levels and higher systolic and diastolic blood pressure. Interestingly, the indeterminate subtype has been associated with lower androstenedione blood levels [39].

Finally, additional studies have suggested that lean PCOS patients present a different genetic pattern to obese PCOS patients [40]. Lean patients were characterized by DENND1A, XBP1 (X-box binding protein 1), and LINCO02905 (long intergenic non-protein-coding RNA 2905) alterations while no specific genetic pattern was observed in obese patients. However, combining overweight and obese patients, a genetic pattern characterized by *DENND1A* and *ERBB4* gene alterations was found. This study confirms the central role of *DENND1A* gene alterations in PCOS patients but suggests that differences in body weight in individuals with PCOS may also be determined by genetic patterns [40].

Of course, more data and models are needed to confirm whether different genes are needed to develop a specific phenotype of PCOS.

## 6. Environmental and Epigenetic Mechanisms That May Be Important in Pathogenesis of PCOS

It is probable that genetic alterations may explain only a portion of PCOS cases. Other factors have to be involved. These factors may be environmental or epigenetic, transmitted during fetal life or additional genetic alterations that are found only in specific subgroups of patients.

Obesity is probably the most important environmental factor that is associated with the syndrome and may modify the clinical expression of the syndrome [3,28,29]. However, in many countries, most patients with PCOS are not obese [28,29,30], which suggests that obesity is not needed for the evolution of a risk condition to the clinical syndrome, but it probably increases the metabolic risk of the disorder [29,30].

Recently, it has been suggested that oxidative stress in patients with an increased genetic risk may be a contributing factor in the etiology of PCOS [41]. In fact, mitochondrial DNA plays a central role in various metabolic processes, including the biosynthesis of steroid hormones, but is very susceptible to damage caused by oxidative stress [41].

Chronic inflammation (maybe linked to obesity but also to other factors) may be an additional factor contributing to the development of PCOS in individuals with increased risk [42,43,44].

More important may be the epigenetic alterations determined by increased values of AMH or androgens during fetal life [34,45]. In fact, several animal models have illustrated a link between prenatal exposure to androgens or anti-Müllerian hormone and PCOS-like phenotypes in subsequent generations, illustrating epigenetic programming in many individuals with PCOS. In humans, epigenetic changes have been reported in peripheral blood mononuclear cells (PBMCs), adipose tissue, granulosa cells (GCs), and liver from individuals with PCOS.

## 7. Conclusions—Searching for a Unifying Hypothesis on Etiologic Mechanisms Determining PCOS

The etiology of PCOS is still to be defined and may be different among subgroups of patients.

In some patients, the contemporaneous presence of several genetic alterations involving some reproductive and metabolic functions (androgen production, follicle regulation, and insulin activity and production) may be sufficient for determining the syndrome. These altered genes may also be rare gene variants that regulate androgen secretion and/or follicle growth.

In many other patients, PCOS appears in women who present a genetic risk for developing the syndrome, but the disorder requires some other nongenetic factors, which may be epigenetic (increased AMH and/or androgens during fetal life) or environmental (for example, obesity or exposure to oxidative stress).

**Figure 1:**
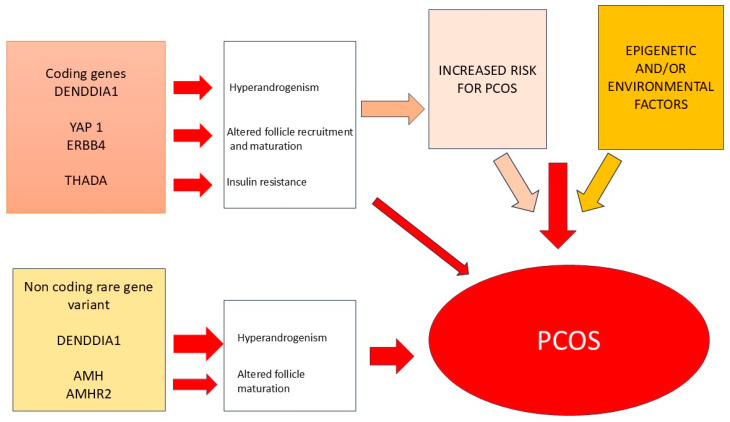
Possible mechanisms causing PCOS.

In Figure 1, the possible genetic mechanisms causing PCOS or an increased risk for developing this syndrome are represented.

Significant progress has been made, and the enigma of the etiology of PCOS (or of different PCOSs) finally seems close to being solved. However, some additional steps are needed, and we need additional studies for an understanding of the interplay between the genetic and non-genetic factors involved in the pathogenesis of this important and common disorder.

## Figures and Tables

**Table 1:** Distribution in different populations of women with PCOS of most common gene alterations found by GWASs.

Gene Alterations	European Ancestry Populations	Chinese Han Population
*DENDD1A*	x	x
*THADA*	x	x
*YAP1*	x	x
*FSHB*	x	
*ERBB4*	x	
